# LncRNA PTCSC3 inhibits cell proliferation in laryngeal squamous cell carcinoma by down-regulating lncRNA HOTAIR

**DOI:** 10.1042/BSR20182362

**Published:** 2019-06-28

**Authors:** Dong Xiao, Xiangyan Cui, Xin Wang

**Affiliations:** Department of Otolaryngology, The First Hospital, Jilin University, Changchun City 130021, Jilin Province, P.R. China

**Keywords:** laryngeal squamous cell carcinoma, lncRNA PTCSC3, lncRNA HOTAIR, proliferation

## Abstract

It is known that lncRNA PTCSC3 inhibits thyroid cancer and glioma and STAT3 promotes cancer development. We, in the present study, investigated the potential involvement of PTCSC3 in laryngeal squamous cell carcinoma (LSCC) and explored its interactions with STAT3. In the present study, we showed that plasma PTCSC3 was down-regulated in early stage LSCC patients, and the down-regulation of PTCSC3 separated in early stage LSCC patients from control group. LncRNA HOTAIR was up-regulated in early stage LSCC patients and was significantly and inversely correlated with PTCSC3 in LSCC patients. PTCSC3 overexpression led to the inhibition of HOTAIR, while PTCSC3 expression was not significantly affected by HOTAIR overexpression. PTCSC3 overexpression mediated the inhibited, while HOTAIR overexpression mediated the promoted proliferation of LSCC cells. However, cell invasion and migration were not significantly affected by PTCSC3 overexpression. In addition, HOTAIR overexpression reduced the inhibitory effects of PTCSC3 overexpression on cancer cell proliferation. Moreover, PTCSC3 overexpression mediated the down-regulation of STAT3 and STAT3 overexpression mediated the up-regulation of HOTAIR. Therefore, PTCSC3 may negatively interact with HOTAIR through STAT3 to inhibit LSCC cell proliferation.

## Introduction

Human genome annotation revealed that approximately 90% of the human genome can be transcribed, while only 2% of the transcripts are related to protein-coding genes, indicating the majority of human genome transcripts are actually non-coding RNAs [1,2]. Long (>200 nt) non-coding RNAs, or LncRNAs, are functional RNAs with pivotal roles in diverse biological procedures by spatially and temporally regulating gene expression [3,4]. LncRNAs are also critical determinants in human diseases and regulation of lncRNAs expression may be beneficial for disease control or treatment [5]. However, functionality of most lncRNAs remains unclear, which hinders the clinical applications of lncRNAs.

Laryngeal squamous cell carcinoma, or LSCC is a common type of cancer and it is also the second most common head and neck malignancy [6,7]. LSCC patients usually suffer from higher morbidity and mortality rates even after active treatment [8]. Especially for patients at advanced stages, the mortality rate is extremely high [9]. Therefore, early diagnosis followed by appropriate treatment is still the key for survival of patients with LSCC. LncRNA PTCSC3 has been characterized as a tumor suppressor lncRNA in thyroid cancer and glioma [10–12]. We performed preliminary analysis (deep sequencing-based transcriptome analysis) and found that PTCSC3 was down-regulated in LSCC and negatively correlated with HOTAIR (data not shown), which is an oncogenic lncRNA in cancer [13]. We therefore investigate the interaction between PTCSC3 and HOTAIR in LSCC.

## Materials and methods

### Research subjects

A total of 66 LSCC patients (gender: 37 males and 29 females; mean age: 48.2 ± 6.6 years) as well as 52 healthy volunteers (gender: 30 males and 22 females; mean age: 47.4 ± 5.2 years) were enrolled in The First Hospital, Jilin University from January 2015 to January 2018. Inclusion criteria of patients: (i) newly diagnosed patients with LSCC; (ii) patients at early stages, which are AJCC stages I and II; (iii) patients who were diagnosed for the first time and received no treatment before admission; (iv) patients who fully understood the experimental procedure and principle and were willing to participate. Exclusion criteria: (i) patients who were transferred from other hospitals or had been treated before admission; (ii) patients who were diagnosed with multiple diseases, such as other types of cancer and severe systemic infections. According to AJCC staging, there were 28 and 38 cases at stages I and II, respectively. The 52 healthy volunteers were selected from Physical Health Center of The First Hospital, Jilin University to match the age and gender distributions of LSCC patients. All 52 healthy volunteers had all physical indicators within normal ranges. The present study has been approved by Ethics Committee of The First Hospital, Jilin University before the admission of patients. World Medical Association Declaration of Helsinki was followed to perform all experiments. Informed consent was provided by all patients and controls.

### Plasma and cell line

Fasting blood (5 ml) was extracted from each subject before therapies. Blood was transferred to an EDTA-treated tube and blood cells are removed and plasma was retained by centrifugation for at 1200×***g*** in a refrigerated centrifuge for 10 min.

UM-SCC-17A human LSCC cell line (Sigma–Aldrich, U.S.A.) was used. Eagle’s Minimum Essential Medium (10% FBS) was the cell culture medium and cells were cultivated in a 5% CO_2_ incubator at 37°C.

### RT-qPCR

RNAzol Reagent (Sigma–Aldrich) was directly mixed with plasma or *in vitro* cultivated cells to extract total RNA. cDNA was prepared using ReadyScript® cDNA Synthesis kit (Sigma–Aldrich) through following conditions: 25°C for 5 min, 53°C for 10 min and 75°C for 10 min. RT-qPCR were performed to detect the expression of PTCSC3 and HOTAIR with all PCR mixtures made using SYBR® Green Quantitative RT-qPCR Kit (Sigma–Aldrich). Primers of PTCSC3 and HOTAIR as well as endogenous control GAPDH were designed and synthesized by Sangon (Shanghai, China). Primer sequences were: 5′-GGCTTGAACAATCTTCCCACCT-3′ (forward) and 5′-TTTGGCAACACCCTCACAGACA-3′ (reverse) for PTCSC3; 5′-CACGGTGCCAGAGAAGAAG-3′ (forward) and 5′-GGGAAGAATCACGCCTTCT-3′ (reverse) for STAT3; 5′-CAGTGGGGAACTCTGACTC-3′ (forward) and 5′-GTGCCTGGTGCTGTCTTAC-3′ (reverse) for HOTAIR; 5′-AAGGTGAAGGTCGGAGTCAA-3′ (forward) and 5′-GGGTCATTGATGGCAACAAT-3′ (reverse) for GAPDH. According to 2^−ΔΔ*C*^_T_ method, expression of PTCSC3 and HOTAIR was normalized to endogenous control GAPDH.

### Cell transfection

PTCSC3, STAT3 and HOTAIR expression vectors were constructed by GenePharma (Shanghai, China) using pcDNA3.1 mammalian cell expression vector. UM-SCC-17A cells were collected at a confluence of 70–80%. Lipofectamine 2000 reagent (Invitrogen, U.S.A.) was used to transfect 10 nM PTCSC3, STAT3 and HOTAIR expression vector or 10 nM empty pcDNA3.1 vector (negative control, NC) into SCC-17A cells. Controls (C) were cells without transfections. Following experiments were carried out at 24 h post-transfection.

### *In vitro* cell proliferation assay

At 24 h post-transfection, UM-SCC-17A cells were harvested and 5 × 10^4^ cells were mixed with 1 ml Eagle’s Minimum Essential Medium (10% FBS) to prepare single cell suspensions. Cells were cultivated under 5% CO_2_ and 37°C in a 96-well plate. Ten microliters CCK-8 solution were added 4 h before the end of cell culture. Following the addition of 10 μl, OD values were measured at 450 nM.

### Western blot

RIPA solution (Sangon, Shanghai, China) was mixed with plasma (1 ml RIPA per 0.15 ml plasma) and SCC-17A cells (1 ml RIPA per 10^5^ cells). Denaturing, electrophoresis (10% SDS/PAGE gel), gel transfer (PVDF membranes), blocking (5% non-fat milk) and blotting were performed in routine manner. Primary antibodies included rabbit polyclonal STAT3 (226943, 1:900, Abcam) and GAPDH (ab9485, 1:900, Abcam). Secondary antibody was IgG-HRP secondary antibody (1:800, goat anti rabbit, MBS435036, MyBioSource). ECL (Sigma–Aldrich, U.S.A.) was used in signal development and ImageJ v1.46 software was used for signal process.

### Statistical analysis

Mean values were calculated using data from three replicates. All comparisons were performed using mean values. Correlations were analyzed by Pearson’s correlation coefficient. Differences between two groups of participants were analyzed using unpaired *t* test. Differences among multiple groups were explored by ANOVA (one-way) and Tukey’s test. Diagnostic analysis was performed using ROC curve. In ROC curve, true positive cases were LSCC patients, and true negative cases were controls. *P*<0.05 was statistically significant.

## Results

### Down-regulation of plasma PTCSC3 distinguished early stage LSCC patients from control group

**Figure 1:**
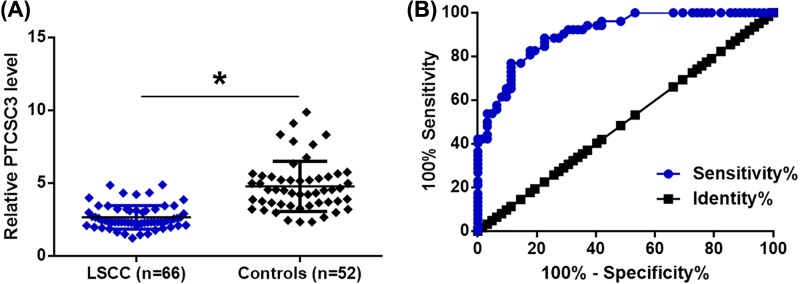
Down-regulation of plasma PTCSC3 distinguished early stage LSCC patients from controls RT-qPCR was performed to detect the expression of PTSCSC2 in LSCC patients and controls (**A**) (*, P<0.05). PCR was repeated three times and data were compared by unpaired t test. ROC curve analysis was performed to evaluate the diagnostic values of PTCSC3 for LSCC (**B**).

Plasma levels of PTCSC3 in 66 LSCC patients and 52 healthy volunteers were measured by RT-qPCR. It was observed that plasma levels of PTCSC3 were significantly lower in LSCC patients compared with control group (Figure 1A, *P*<0.05). Diagnostic values of plasma PTCSC3 for LSCC were evaluated by ROC curve analysis. As shown in Figure 1B, area under the curve was 0.91, with standard error of 0.026 and 95% confidence interval of 0.85–0.96 (Figure 1B).

**Figure 2:**
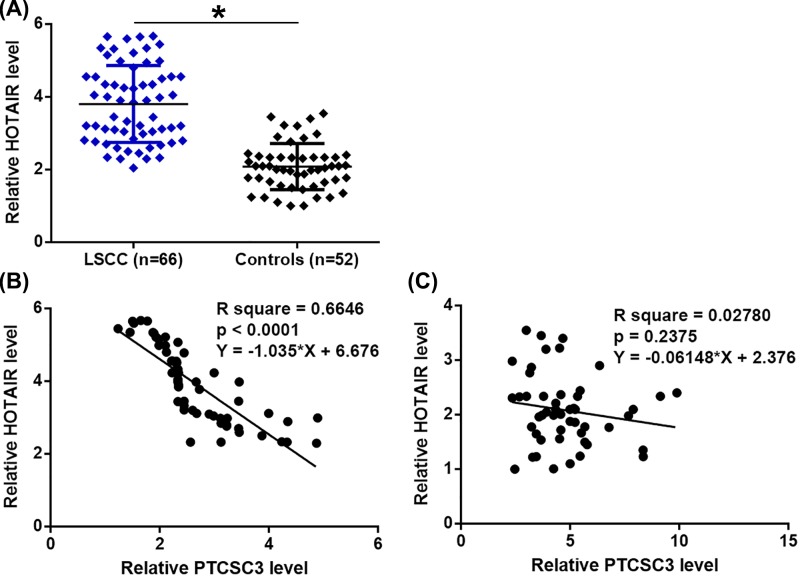
HOTAIR was up-regulated and inversely correlated with PTCSC3 in early stage LSCC patients RT-qPCR was performed to detect the expression of HOTAIR in LSCC patients and controls (**A**) (*, P<0.05). PCR was repeated three times and data were compared by unpaired t test. Pearson’s correlation coefficient was performed to analyze the correlation between HOTAIR and PTCSC3 in LSCC patients (**B**) and controls (**C**).

### HOTAIR was up-regulated and inversely correlated with PTCSC3 in early stage LSCC patients

Plasma levels of HOTAIR in 66 LSCC patients and 52 healthy volunteers were also measured by RT-qPCR. It was observed that plasma levels of HOTAIR were significantly higher in LSCC patients compared with control groups (Figure 2A, *P*<0.05). Pearson’s correlation coefficient showed that HOTAIR and was significantly and inversely correlated with PTCSC3 only in LSCC patients (Figure 2B). In contrast, the correlation between PTCSC3 and HOTAIR was not significant in controls (Figure 2C).

### PTCSC3 is an upstream negative regulator of HOTAIR in LSCC cells

**Figure 3:**
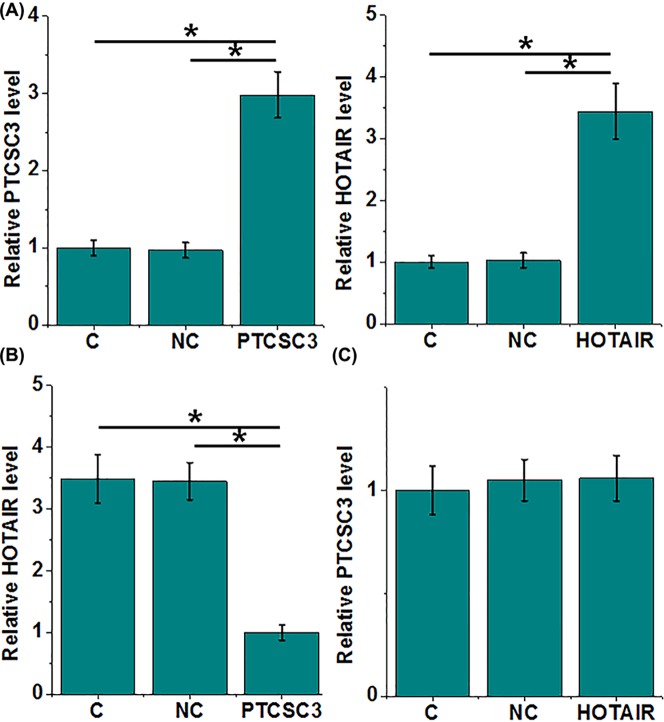
PTCSC3 is an upstream negative regulator of HOTAIR in LSCC cells PTCSC3 and HOTAIR overexpression were were detected at 24 h post-transfection to confirm their overexpression (**A**). The effects of PTCSC3 overexpression on HOTAIR (**B**) and the effects of HOTAIR overexpression on PTCSC3 (**C**) were also analyzed by performing RT-qPCR. Three biological replicates were included and data were compared by ANOVA (one-way) and Tukey’s test, (*, P<0.05).

PTCSC3 and HOTAIR overexpression were performed in cells of UM-SCC-17A cell line (Figure 3A, *P*<0.05). Comparing with control cells (C) and NC cells (NC), overexpression of PTCSC3 led to significantly inhibited expression of HOTAIR in cells of UM-SCC-17A (Figure 3B, *P*<0.05). In addition, PTCSC3 was not significantly affected by HOTAIR overexpression (Figure 3C, *P*<0.05).

### PTCSC3 overexpression mediated the inhibited proliferation of LSCC through HOTAIR

**Figure 4:**
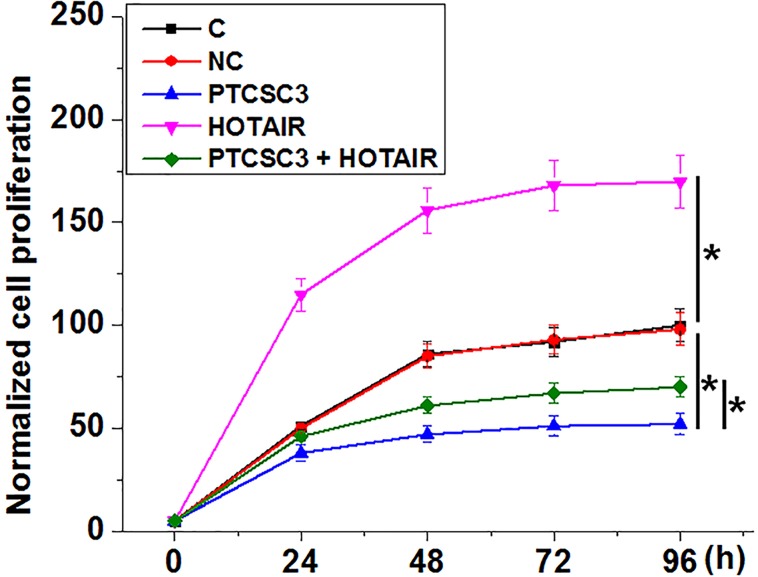
PTCSC3 overexpression mediated the inhibited proliferation of LSCC through HOTAIR Cell proliferation assay was performed at 24 h post-transection. This assay was done in triplicate manner and data were compared by ANOVA (one-way) and Tukey’s test (*, p<0.05).

Comparing with NC and C two controls, groups, PTCSC3 overexpression mediated the inhibited, while HOTAIR overexpression mediated the promoted proliferation of LSCC cells (Figure 4, *P*<0.05). In addition, HOTAIR overexpression attenuated the inhibitory effects of PTCSC3 overexpression on cancer cell proliferation (*P*<0.05). However, cancer cell invasion and migration were not significantly affected by PTCSC3 overexpression (data not shown).

### PTCSC3 down-regulated HOTAIR possible through STAT3

**Figure 5:**
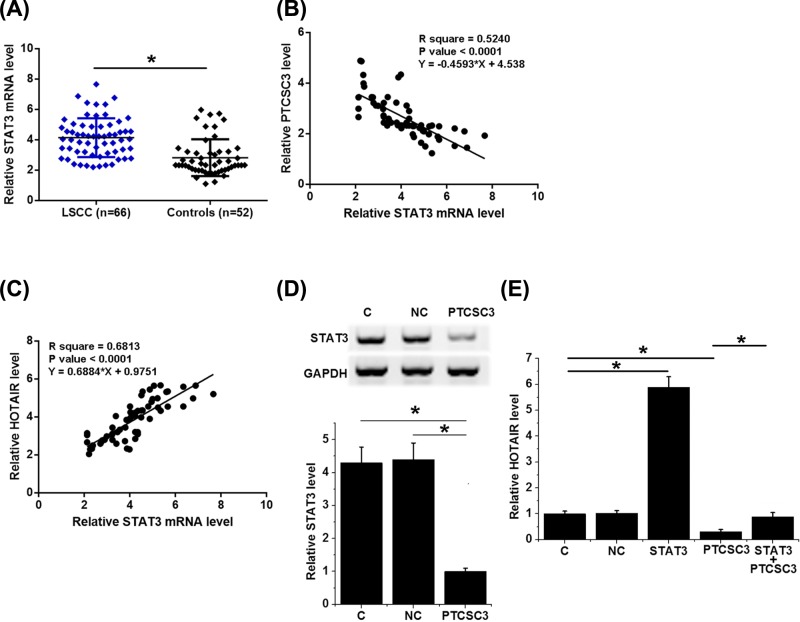
PTCSC3 down-regulated HOTAIR possible through STAT3 Expression levels of STAT3 mRNA were measured by RT-qPCR and compared by unpaired test (**A**). Correlations between STAT3 mRNA and PTCSC3 (**B**)/HOTAIR (**C**) were analyzed by linear regression. Expression of STAT3 (**D**) and HOTAIR (**E**) were detected at 24 h post-transfection by Western blot and RT-qPCR, respectively. This experiment was repeated three times. Data were compared by ANOVA (one-way) and Tukey’s test (*, *P*<0.05).

Expression levels of STAT3 mRNA were measured by RT-qPCR. Comparing with controls, expression levels of STAT3 mRNA were significantly higher in LSCC patients (Figure 5A, *P*<0.05). Correlation analysis showed that STAT3 mRNA was significantly and inversely correlated with PTCSC3 (Figure 5B, *P*<0.05), but was significantly and positively correlated with HOTAIR (Figure 5C, *P*<0.05). Comparing with NC and C two controls, overexpression of PTCSC3 led to down-regulated expression of STAT3 in LSCC cells (Figure 5D, *P*<0.05). In addition, comparing with the two controls, STAT3 resulted in the up-regulation of HOTAIR and attenuated the effects of PTCSC3 overexpression on HOTAIR (Figure 5E, *P*<0.05).

## Discussion

LSCC is characterized by the unacceptable high mortality rate, and novel early diagnosis techniques are urgently needed. We found that PTCSC3 was down-regulated in LSCC patients and may serve as a biomarker for the early diagnosis of LSCC.

HOTAIR as a master regulator of chromatin dynamics is a well-characterized oncogenic lncRNAs in different types of human cancer [13]. In effect, down-regulation of HOTAIR provides new insights for cancer treatment [14]. HOTAIR is overexpressed in LSCC [15]. Consistently, we also observed up-regulated HOTAIR expression in plasma of LSCC patients. HOTAIR promotes tumor progression through the interactions with multiple downstream signaling pathways, such as tumor suppressor PTEN [15]. However, the upstream regulators of HOTAIR are largely unknown. We proved that PTCSC3 as a formerly characterized tumor suppressor in thyroid cancer and glioma [10–12] was likely an upstream inhibitor of HOTAIR, and the inhibition of HOTAIR by PTCSC3 is involved in the regulation of LSCC cell proliferation. Our date enriched our understanding on the functionality of HOTAIR in cancer biology and suggested that overexpression of PTCSC3 may serve as a therapeutic target for LSCC.

It is known that suppression of STAT3 expression is associated with concomitant down-regulation of HOTAIR expression [16], while PTCSC3 inhibits STAT3 expression [17]. We found that in LSCC cells PTCSC3 down-regulated the expression of both STAT3 and HOTAIR and STAT3 up-regulated HOTAIR. Therefore, STAT3 may mediate the interaction between PTCSC3 and HOTAIR. However, the mechanism of the down-regulation of STAT3 by PTCSC3 is unknown. Future studies are needed.

Interestingly, our study observed that PTCSC3 overexpression led to inhibited LSCC cell proliferation, while cell invasion and migration were not significantly affected by PTCSC3 (data not shown). It is clear that HOTAIR promotes cancer cell migration and invasion [18,19]. Therefore, PTCSC3 as the upstream inhibitor of HOTAIR should also interact with other factors to inhibit the inhibitor effects of HOTAIR down-regulation (as a result of PTCSC3 overexpression) on cancer cell migration and invasion. In effect, PTCSC3 should also interact with multiple factors to regulate LSCC cell proliferation because HOTAIR overexpression only partially rescued the inhibitory effect of PTCSC3 overexpression on cancer cell proliferation. However, more studies, especially *in vivo* animal model experiments are needed to further validate our observations and conclusion.

In conclusion, PTCSC3 is down-regulated in LSCC and PTCSC3 overexpression may PTCSC3 may down-regulate HOTAIR to inhibit LSCC cell proliferation.

## Abbreviations

lncRNA: long non-coding RNA
LSCC: laryngeal squamous cell carcinoma
NC: negative control
